# Probiotics as Adjuvant Treatment for Psychiatric Disorders: A Systematic Review

**DOI:** 10.1192/j.eurpsy.2023.558

**Published:** 2023-07-19

**Authors:** E. A. Forth, B. Buehner, A. Storer, C. Sgarbossa, R. Milev, A. Chinna Meyyappan

**Affiliations:** ^1^ Centre for Neuroscience Studies; ^2^Department of Psychiatry, Queen’s University; ^3^Department of Psychiatry, Providence Care Hospital; ^4^Department of Psychology, Queen’s University, Kingston, Canada

## Abstract

**Introduction:**

Many psychiatric illnesses have been linked to the gut microbiome, with supplements such as probiotics showing some efficacy in alleviating the symptoms of some psychiatric illnesses. Though probiotics alone have been found to be efficacious in alleviating the symptoms of psychiatric illnesses, the combination of probiotics and first-line psychotropic medications has not been investigated as thoroughly.

**Objectives:**

The primary objective of this review was to evaluate the current literature investigating the effects of adjuvant probiotic or synbiotic administration in combination with first-line psychotropic treatments for psychiatric illnesses.

**Methods:**

A systematic search of four databases was conducted using key terms related to treatments for psychiatric illnesses, the gut microbiome, and probiotics. All results were then evaluated based on specific eligibility criteria. The salient outcome measures from the studies that met this eligibility criteria were then extracted and analysed.

**Results:**

Eight studies met eligibility criteria and were analysed for reported changes in outcome measures used to assess the symptoms of psychiatric illness and the tolerability of treatment. All Major Depressive Disorder (MDD) (n=5) and Generalized Anxiety Disorder (GAD) (n=1) studies found adjuvant probiotic or synbiotic treatment to be more efficacious in improving the symptoms of psychiatric illness than the first-line treatment alone or with placebo. The schizophrenia studies (n=2) found adjuvant probiotic treatment to have no significant difference in clinical outcomes, but it was found to improve the tolerability of first-line antipsychotics.

**Conclusions:**

The findings of the studies included in this review suggest the use of adjuvant probiotic treatment with selective serotonin reuptake inhibitors (SSRIs) for MDD and GAD to be superior to SSRI treatment alone. Probiotic adjuvant treatment with antipsychotics could be beneficial for improving the tolerability of the antipsychotics, but these findings do not suggest that adjuvant probiotic treatment would result in improved clinical outcomes for symptoms of schizophrenia.

**Disclosure of Interest:**

None Declared

